# Automated radiomics model for prediction of therapy response and minimal residual disease from baseline MRI in multiple myeloma

**DOI:** 10.1038/s41598-025-13165-2

**Published:** 2025-10-10

**Authors:** Fabian Bauer, Marina Hajiyianni, Niels Weinhold, Martin Grözinger, Jessica Kächele, Ekaterina Menis, Marc-Steffen Raab, Sandra Sauer, Anna Jauch, Tim F. Weber, Manuel Debic, Britta Besemer, Marius Horger, Saif Afat, Martin Hoffmann, Johannes Hoffend, Doris Kraemer, Ullrich Graeven, Adrian Ringelstein, Jan Dürig, Lale Umutlu, Heinz-Peter Schlemmer, Hartmut Goldschmidt, Klaus Maier-Hein, Stefan Delorme, Elias K. Mai, Markus Wennmann, Peter Neher

**Affiliations:** 1 Division of Radiology, German Cancer Research Center (DKFZ), Im Neuenheimer Feld 280, 69120 Heidelberg, Germany; 2 Division of Musculoskeletal Imaging and Intervention, Department of Radiology, Massachusetts General Hospital, Harvard Medical School, Boston, MA 02114 USA; 3 Institute for Diagnostic and Interventional Radiology, Faculty of Medicine and University Hospital Cologne, University of Cologne, 50937 Cologne, Germany; 4 Internal Medicine V, Hematology, Oncology and Rheumatology, Heidelberg University Hospital, 69120 Heidelberg, Germany; 5 Division of Medical Image Computing, German Cancer Research Center (DKFZ), 69120 Heidelberg, Germany; 6 German Cancer Consortium (DKTK), Partner Site Heidelberg, 69120 Heidelberg, Germany; 7 Institute of Human Genetics, University of Heidelberg, 69120 Heidelberg, Germany; 8 Diagnostic and Interventional Radiology, University Hospital Heidelberg, 69120 Heidelberg, Germany; 9 Department of Hematology, Oncology, and Immunology, University Hospital Tuebingen, 72016 Tübingen, Germany; 10 Department of Diagnostic and Interventional Radiology, University Hospital Tuebingen, 72016 Tübingen, Germany; 11 Medical Clinic A, Ludwigshafen Clinical Center, 67063 Ludwigshafen, Germany; 12 Department of Radiology, Ludwigshafen Clinical Center, 67063 Ludwigshafen, Germany; 13 Department of Hematology, Oncology and Palliative Care, St. Josefs Hospital Hagen, 58097 Hagen, Germany; 14 Department of Hematology, Oncology, and Gastroenterology, Kliniken Maria Hilf GmbH, Mönchengladbach, Germany; 15 Department of Radiology and Neuroradiology, Kliniken Maria Hilf GmbH, 41063 Mönchengladbach, Germany; 16 MVZ Essen-Nord, St. Augustinus MVZ GmbH, Gelsenkirchen, Germany; 17 Department of Diagnostic and Interventional Radiology and Neuroradiology, University Hospital Essen, 45141 Essen, Germany; 18 National Center for Tumor Diseases (NCT), University Hospital Heidelberg, 69120 Heidelberg, Germany; 19 Pattern Analysis and Learning Group, Department of Radiation Oncology, Heidelberg University Hospital, 69120 Heidelberg, Germany

**Keywords:** Multiple myeloma, Radiomics, MRI, Therapy response, Minimal residual disease., Bone cancer, Myeloma

## Abstract

This multicenter imaging study aimed to establish and validate automated radiomics models predicting therapy response (TR) and minimal residual response (MRD) in newly diagnosed multiple myeloma (MM) from baseline MRI. Retrospectively, 118 MM patients from the GMMG-HD7 trial (EudraCT: 2017-004768-37) with data on TR and/or MRD after induction therapy and baseline MRI were included. Data were split by center into a training set (center 1–2; *n* = 79) and a test set (center 3–10; *n* = 39). TR was classified as very good partial response or better versus other. An in-house developed nnU-Net was used to automatically segment pelvic bone marrow for the subsequent extraction of 245 radiomics features and piriformis muscle for normalization. Random forest classifiers were trained using radiomics features only (I), radiomics features with additional confounders (II) or myeloma-relevant clinical features (III), or only clinical features (IV) to predict TR or MRD status. The area under the receiver operating characteristic curve (AUROC) was calculated to assess prediction performance. The prediction model using only radiomics features (I) showed the highest predictive performance for TR on the test set with an AUROC of 0.70. AUROC values for radiomics-based prediction of the MRD status (I-III) ranged from 0.54 to 0.52. In conclusion, our study demonstrated the potential of automated radiomics models from baseline MRI to non-invasively predict TR in MM on an independent, multicentric test set.

**Supplementary Information:**

The online version contains supplementary material available at 10.1038/s41598-025-13165-2.

## Introduction

The clinical presentation of multiple myeloma (MM) can vary widely in terms of tumor burden, symptoms, tumor genetics, and outcome^1^. Newly diagnosed multiple myeloma (NDMM), as defined by the International Myeloma Working Group (IMWG), typically warrants therapy^2^. Eligible NDMM patients are often recommended intensive, high-dose treatment regimens followed by autologous transplantation^3^. Therapy response (TR), and occasionally the possible presence of minimal residual disease (MRD), is assessed after the administration of induction therapy^4^ and serve as early predictors for long-term outcome in MM^5–8^. TR is usually categorized based on serum or urine monoclonal protein (M-protein) or the serum free light chain ratio (SFLCR), and plasma cell infiltration (PCI) of the bone marrow (BM) according to the IMWG criteria. Next-generation sequencing (NGS) and next-generation flow (NGF) are used to determine MRD status and provide an estimate of the number of residual myeloma cells with a required minimum sensitivity of 1 in 10^5^ nucleated cells^4^. Providing an accurate prognostic assessment for NDMM patients remains challenging^1^despite recent advances in treatment and overall patient outcome^9,10^. Non-invasive prediction of TR and MRD at baseline could potentially improve clinical decision making.

Whole-body MRI (WBMRI) provides comprehensive information on the BM status in MM^11^capturing the spatial heterogeneity of tumor manifestation^12,13^. Therefore, WBMRI allows the detection of diffuse infiltration pattern or focal lesions (FL)^11^. These features are of prognostic value, as shown for the presence of diffuse infiltration^14,15^ and FLs^16,17^ in NDMM, as well as for the detection of FLs during treatment assessment in MM^18,19^. The IMWG recommends WBMRI for baseline imaging^20^and both TR and MRD assessment can be complemented by WBMRI, which allows tracking of new and residual FLs^21^. Radiomic features encode high-dimensional image characteristics based on shape, signal intensity, or texture of the volume of interest^22^. Radiomics features, either on their own or in combination with clinical data, can be utilized to predict outcomes or histologic results and provide additional information about tumor characteristics^23,24^.

Previous radiomics studies in MM have provided valuable insights into predicting TR^25,26^ and MRD^27^ from baseline MRI. However, many of these important contributions have been based on single-center cohorts and have not yet included external validation, which may limit their generalizability and hinder clinical applicability. In addition, the manual or semi-automatic segmentation method of most existing pipelines introduces inter-observer variability and further constrains scalability, particularly in resource-limited settings. A fully automated, generalizable radiomics pipeline may overcome these limitations by improving reproducibility, reducing manual workload, and supporting large-scale implementation in clinical settings.

The aim of this study was to establish a fully automated machine learning model capable of predicting TR and MRD from baseline MRI, complemented by baseline clinical features, and to test the developed models on multicenter imaging data.

## Materials and methods

For this retrospective multicenter study, appropriate ethical approval from the institutional review board was obtained (S-537/2020, clinical trial number: not applicable) with informed consent being waived. This study was performed in accordance with the Declaration of Helsinki and adhered to the relevant guidelines and regulations of our institution.

### Study cohort

**Fig. 1:**
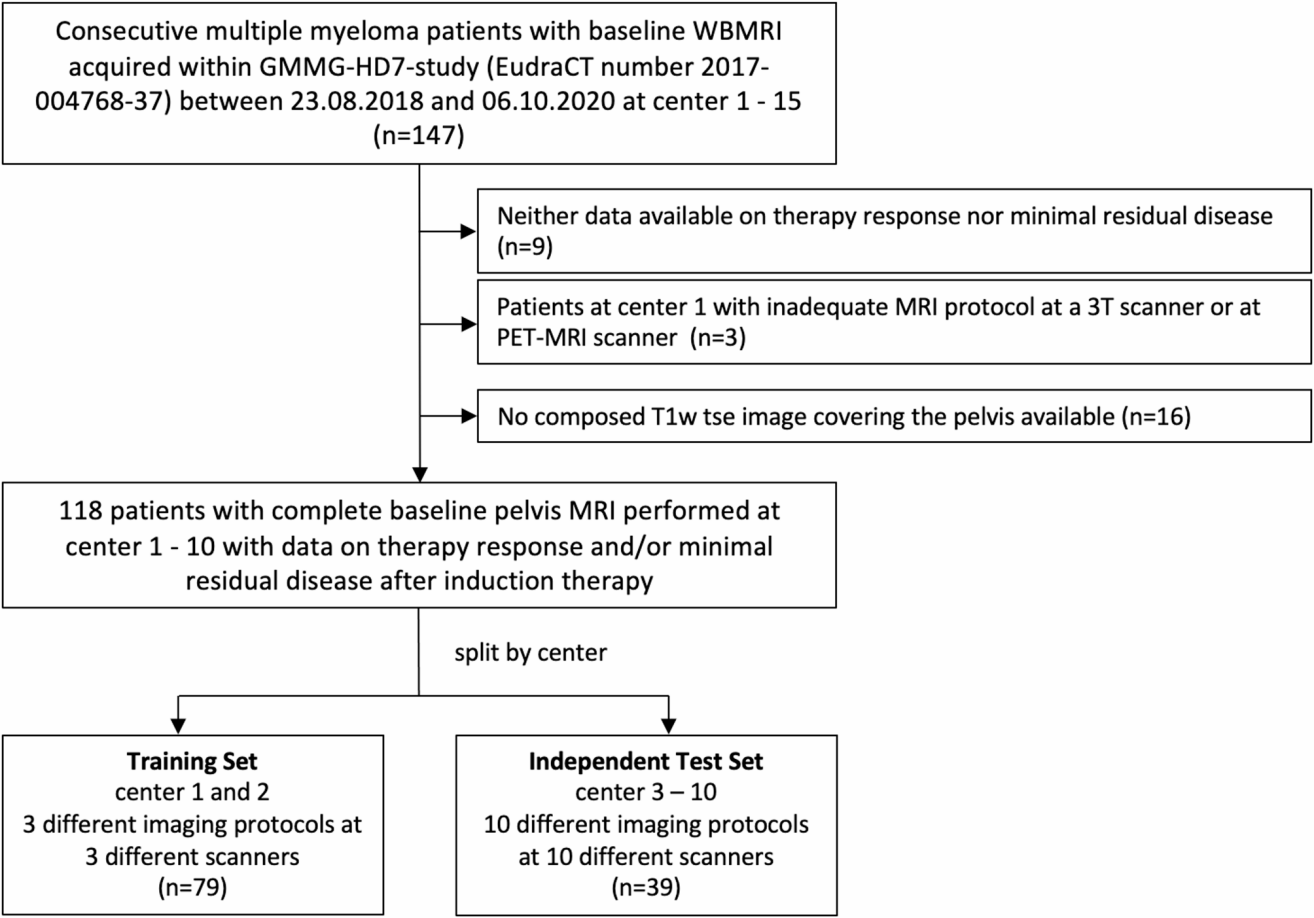
Flow chart. Baseline T1-weighted turbo spin echo sequence (T1w tse) MRIs with corresponding information on therapy response and/or minimal residual disease status after induction therapy from center 1 and 2 of the GMMG-HD7 trial were used in the training set. The independent, external, multicentric test set included baseline MRIs from center 3–10 with MRI scanners from various vendors and with different protocols.

Unenhanced WBMRIs and clinical data were acquired within the phase 3 GMMG-HD7 trial (EudraCT: 2017-004768-37) of the German Speaking Myeloma Multicenter Group (GMMG) between 2018 and 2020 as well as 2021, respectively. Detailed inclusion criteria have been reported by Goldschmidt et al. in 2022^28^. In addition, inclusion criteria in this study were a complete pelvis MRI performed before treatment start and availability of data on either or both MRD and TR status after induction. The GMMG-HD7 cohort with unenhanced baseline pelvis MRIs and corresponding clinical data was split by the location of the imaging centers. Data from centers 1 and 2 were used to train the machine learning classification models. Data from the centers 3–10 were included in the independent, external test set, which provided very heterogeneous testing conditions due to the heterogenous image acquisition settings of the included imaging data included. The respective flow chart is shown in Fig. 1. The imaging data has been used partly within different study cohorts in other studies from our institution^28–31^.

### Imaging

In this study, unenhanced coronal T1w turbo spin echo pelvis MRIs were used, which were acquired within the baseline WBMRIs up to 6 weeks prior to the start of therapy. Imaging was performed at different MRI scanners with diverse sequences from several vendors at multiple imaging centers. The MRI acquisition parameters can be found in Supplementary Table S1.

### Clinical data collection

Clinical data used in this study was collected within the multicenter, randomized, active-controlled, phase 3 GMMG-HD7 trial with MRD status as the primary endpoint of the first part of the study, which has been reported elsewhere^28^. Baseline clinical data and MRIs were collected at study entry before therapy. Patients were assigned randomly to undergo three cycles of induction therapy, either with isatuximab in addition to lenalidomide, bortezomib, and dexamethasone or with lenalidomide, bortezomib, and dexamethasone alone. Treatment was assessed within 7 days after completion of the induction therapy using the IMWG criteria for TR and MRD status. TR included complete response (CR), very good partial response (VGPR), partial response, minimal response, stable disease, progressive disease^4^ and near-complete response (nCR), which was established as an additional class. MRD was evaluated using multiparametric NGF at a sensitivity cutoff of 1 tumor cell × 10⁵ nucleated cells^28^. Relevant clinical parameter collected before treatment included patient’s age, sex, body mass index (BMI), M-protein, SFLC-ratio, beta2-microglobulin, calcium levels, creatinine, lactate dehydrogenase (LDH), serum albumin, serum total protein, hemoglobulin, PCI, major histocompatibility complex (MHC) type, del(17p), gain(1q), t(4;14), and the treatment arm^2,28,32,33^. PCI was defined as the higher value of the histological or cytological derived PCI percentage in alignment with IMWG recommendations^2^. Cytogenetic aberrations were marked as clinical parameter by analogy to the R2-ISS criteria^33^. All clinical parameters were used as individual clinical features. Age, BMI and the treatment arm were marked as clinical confounders, since age and BMI are known to influence the BM signal^34,35^. Within the treatment regime of the GMMG-HD7 study, Isatuximab was associated with an elevated proportion of MRD-negative patients^28^.

### Algorithmic architecture

**Fig. 2:**
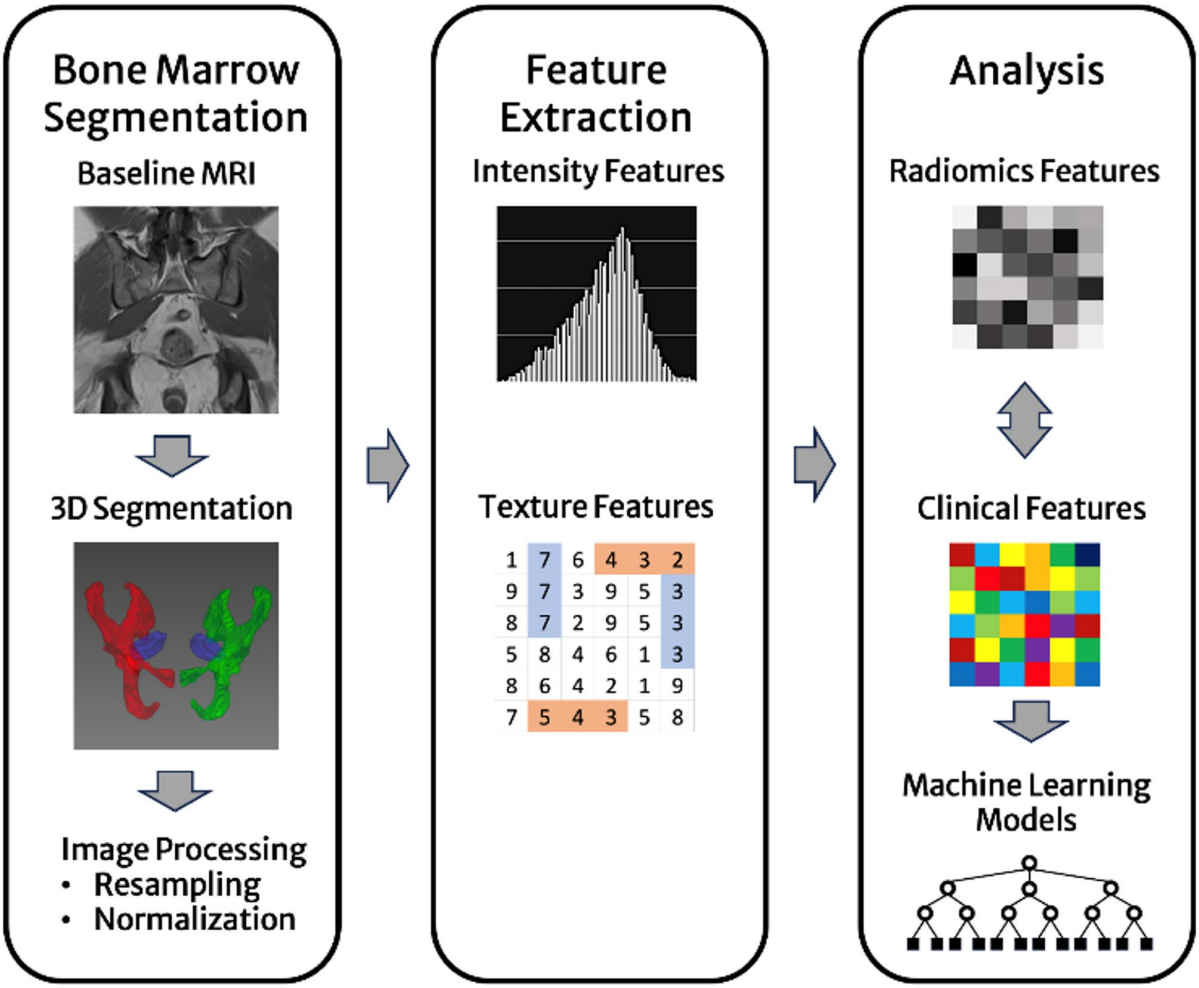
Algorithmic concept of the study. In step I, the bone marrow of the pelvis and the piriformis muscle are automatically being segmented in the coronal T1-weighted turbo spin echo sequence by a previously trained nnU-Net and the bone marrow of each hip bone is individually labeled. Images are normalized to the mean signal intensity of the piriformis muscle and geometrically resampled. In step II, radiomics intensity and texture features are calculated from the pelvic bone marrow. In step III, radiomics and clinical features are utilized in machine learning models to predict therapy response and minimal residual disease status.

Figure 2 displays the algorithmic architecture of the study. A previously in-house-developed and externally validated nnU-Net segmentation algorithm was used to automatically segment the left and right pelvic BM, excluding the cortical bone, and the medial portion of the piriformis muscle on T1w images^31^.

Prior to feature extraction, all MR images were resampled to a uniform voxel spacing that corresponded to the acquisition protocol of center 1, which served as the reference center due to its high image quality, representative acquisition parameters, and the largest group of patients having received a scan at center 1. This resampling step was applied to both training and external test datasets to reduce variability in spatial resolution across scanners and institutions. Following resampling, all images in both the test and training cohort underwent subject-specific intensity normalization using the mean signal intensity of the bilateral piriformis muscle tissue in each scan, following prior work^31^. This biologically grounded normalization approach accounts for inter-scanner variability by scaling each patient’s bone marrow signal to the internal reference tissue. Muscle-based normalization has been shown to improve inter-scanner comparability^36^ and enhances the repeatability and reproducibility of radiomics features across varying acquisition parameters^37^. No additional batch effect correction or statistical harmonization was applied to avoid potential information leakage between the training and independent test sets.

Radiomics first-order and texture features were calculated with the publicly available software MITK Phenotyping^38^. Radiomics features were computed individually for each hip bone, and the mean value was used for further analysis. Radiomics features with zero variance were excluded. Clinical features were also included in this study in alignment with the Radiomics Quality Score^39^. Missing clinical values were encoded as −1. Categorial variables were transformed using one-hot encoding^40^. The primary objective in this study was to predict TR, which was binarily classified as CR, nCR or VGPR versus worse response categories of therapy assessment, in line with the categorization within the GMMG-HD7 trial^28^. MRD negativity was the second target parameter to be predicted independently from TR. Four different random forest classifiers (RFC) were trained to predict each of the two target parameters MRD status and TR based on radiomics features only (I), radiomics features and clinical confounders (II), radiomics and clinical features (III), and clinical features only (IV). While prior work has identified subsets of radiomics features with high reproducibility across scanners^37^ all radiomics features were used in alignment with recent findings showing that RFCs trained on all features achieved superior performance for MRI-based prediction of clinical parameters in MM^41^. The RFC was chosen for its robustness in handling high-dimensional, heterogeneous data and its intrinsic ability to reduce overfitting by combining multiple trees in an ensemble, training each tree on a random subset of the data, and considering only a random subset of features for splitting at each node, which limits the ability of any single tree to overfit the data. This approach not only enhances generalizability but also provides built-in feature importance metrics, improving model interpretability. For the RFCs, default parameters were used with n_estimators = 10,000 and random_state = 0. All machine learning modeling was performed with Python 3.11.6 (Python Software Foundation, Wilmington, Delaware, USA), module scikit-learn 1.1.3^40^. The prediction models were tested on the external, multicentric test set. The METhodological RadiomICs Score (METRICS)^42^ and the CheckList for EvaluAtion of Radiomics research (CLEAR)^43^ results are reported in Supplementary Tables S2 and S3.

### Statistical analysis

The area under the receiver operating characteristic (AUROC) with a 95% confidence interval (95%-CI) was calculated to assess the performance of each prediction model. 95%-CIs were calculated following DeLong et al.^44^. The Youden index was calculated to define the optimal cutoff to calculate sensitivity, specificity, and F1 score. *P* <.05 were considered statistically significant. The Gini feature importance was used to report the relative influence of a radiomics feature on the prediction model. The statistical analysis was performed with Python version 3.11.6, modules scikit-learn^40^ matplotlib version 3.8.3^45^ and seaborn version 0.13.2^46^.

## Results

### Study cohort

Patient characteristics and MM-related parameters are shown for the training set and test set in Table 1. One hundred eighteen baseline MRIs of 118 patients from 10 imaging centers enrolled in the GMMG-HD7 trial were included. The training set comprised 79 MRIs of 79 patients from 2 centers. 39 MRIs of 39 patients from 8 different imaging centers were included in the test set (Fig. 1). One hundred seventeen MRIs had corresponding information on TR (training set: 78, test set: 39) and 114 MRIs had corresponding information on MRD status (training set: 75, test set: 39). Ninety-five first order and 150 texture radiomics features were used as an input for the RFCs in the prediction models (Supplementary Table S4). For some MRIs, corresponding clinical information was not available (no information: m-protein: 40/118, del17p: 4/118 and t(4;14): 4/118). There were no significant differences in age, BMI, and treatment arm between the training set and the test sets (*p* ≥.38).

**Table 1:** Characteristics of the study cohort.

	Training Set (*n* = 79)	Test Set (*n* = 39)
**Center**	Center 1–2	Center 3–10
**Patient Characteristics**		
Male Sex (n; %)	54; 68%	22; 56%
Age in Years (median; IR)	61; 54–67	60; 52–65
BMI in kg/m^2^ (median; IR)	25.5; 23.7–29.4	26.4; 23.7–30.4
Treatment Arm + Isatuximab (n)	37	17
**Treatment Assessment**		
MRD Negative (n)	38	19
Not Evaluable (n)	4	0
**Treatment Response**		
Very Good Partial Response or better(n)	62	28
Partial Response or worse (n)	16	11
Not Evaluable (n)	1	0
**Clinical Parameters**		
ISS Stadium I/II/III (n)	42/21/16	14/16/9
M-Protein in g/l (median; IR)	34.7; 24.7–43.4	32.6; 17.0–43.3
SFLC-Ratio (median; IR)	61; 12–192	113; 39–281
Beta2-Microglobulin in mg/l (median; IR)	3.1; 2.1–4.7	4.3; 2.6–5.4
Calcium Levels in mmol/l (median; IR)	2.4; 2.2–2.5	2.3; 2.2–2.4
Creatinine in mg/dl (median; IR)	0.86; 0.76–1.04	0.88; 0.71–1.24
LDH in U/l (median; IR)	182; 152–212	190; 153–219
Serum Albumin in g/l (median; IR)	39.6; 37.5–43.8	35.0; 28.3–41.0
Serum Total Protein in g/l (median; IR)	91.0; 76.9–101.7	86.8; 68.5–97.9
Hemoglobulin in g/dl (median; IR)	11.7; 10.4–13.1	10.8; 9.4–12.5
Plasma Cell Infiltration in % (median; IR)	55; 20–70	50; 25–75
Major Histocompatibility Complex Type (n)	50 (IgG)/17 (IgA)/1 (IgM)/10 (Light Chain)/1 (Biclonal)	22 (IgG)/10 (IgA)/0 (IgM)/6 (Light Chain)/1 (Biclonal)
**Cytogenetics**		
gain(1q) (n)	28	10
del(17p) (n)	10	2
t(4;14) (n)	14	3

### Prediction of therapy response

**Fig. 3:**
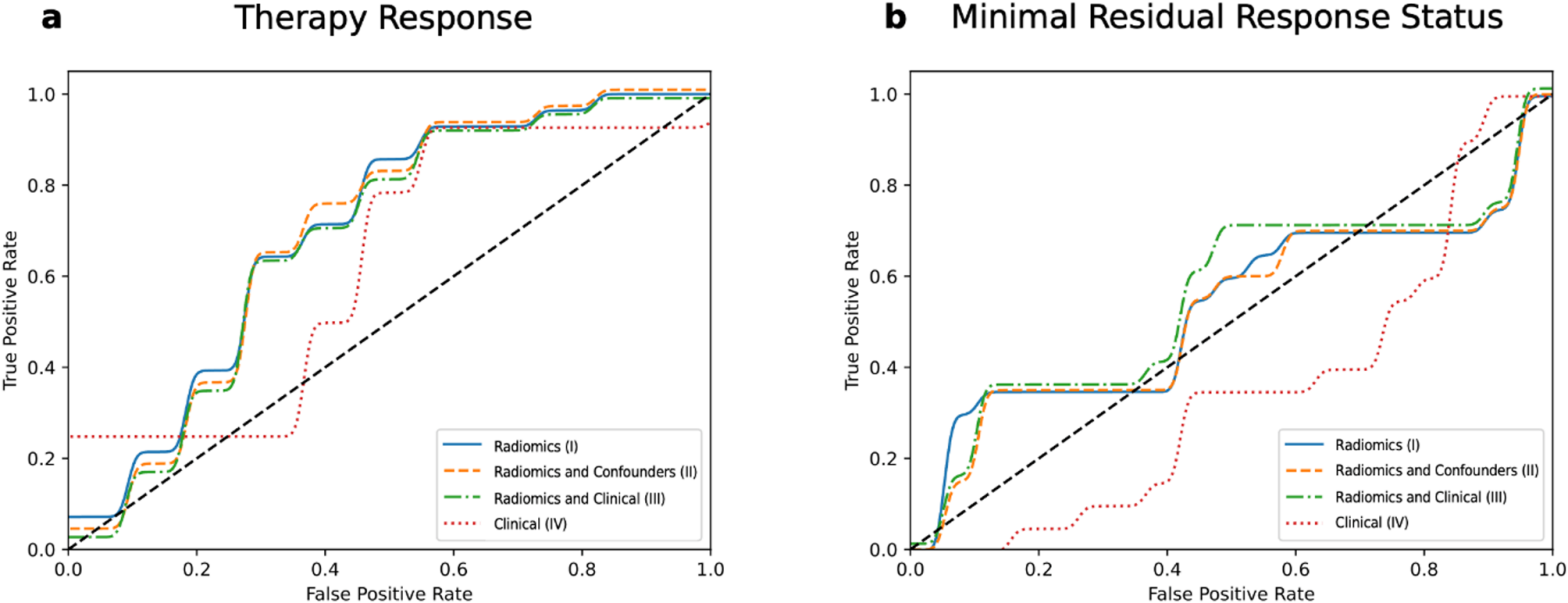
Predictive performance of the different machine learning models. (**a**) ROC values are displayed for the prediction of therapy response of the four different models (I–IV). (**b**) ROC values are shown for the prediction of minimal residual disease status of the four separate machine learning models (I–IV).

Four models were trained for the prediction of binary classified TR. The performance metrics and respective ROC curves are shown in Table 2; Fig. 3. The model based on radiomics features only (I) achieved the best prediction performance for TR on the test set with an AUROC of 0.70. With radiomics models that also included confounders (II) or clinical features (III), the prediction performance was no better than the radiomics features only (I) model (both models with an AUROC of 0.69). For all models that included radiomics features (I-III), the prediction performance in AUROC value for TR was better than for the model using clinical features exclusively (IV; AUROC of 0.63). However, this tendency was not statistically significant (*p* ≥.68).

**Table 2:** Prediction performance on the test set. Performance metrics are given for each model and the target variables TR and MRD separately.

	Performance Metrics	Radiomics Features Only Model (I)	Radiomics Features + Confounders Model (II)	Radiomics + Clinical Features Model (III)	Clinical Features Only Model (IV)
**TR**	AUROC (95%CI)	0.70 (0.49–0.91)	0.69 (0.47–0.91)	0.69 (0.47–0.91)	0.63 (0.41–0.85)
	Sensitivity	0.86	0.75	0.93	0.93
	Specificity	0.55	0.64	0.45	0.45
	F1-Score	0.84	0.79	0.87	0.87
**MRD**	AUROC (95%CI)	0.53 (0.34–0.72)	0.52 (0.33–0.71)	0.54 (0.34–0.73)	0.35 (0.17–0.53)
	Sensitivity	0.30	0.35	0.35	1.00
	Specificity	0.95	0.89	0.89	0.11
	F1-Score	0.44	0.48	0.48	0.70

**Fig. 4:**
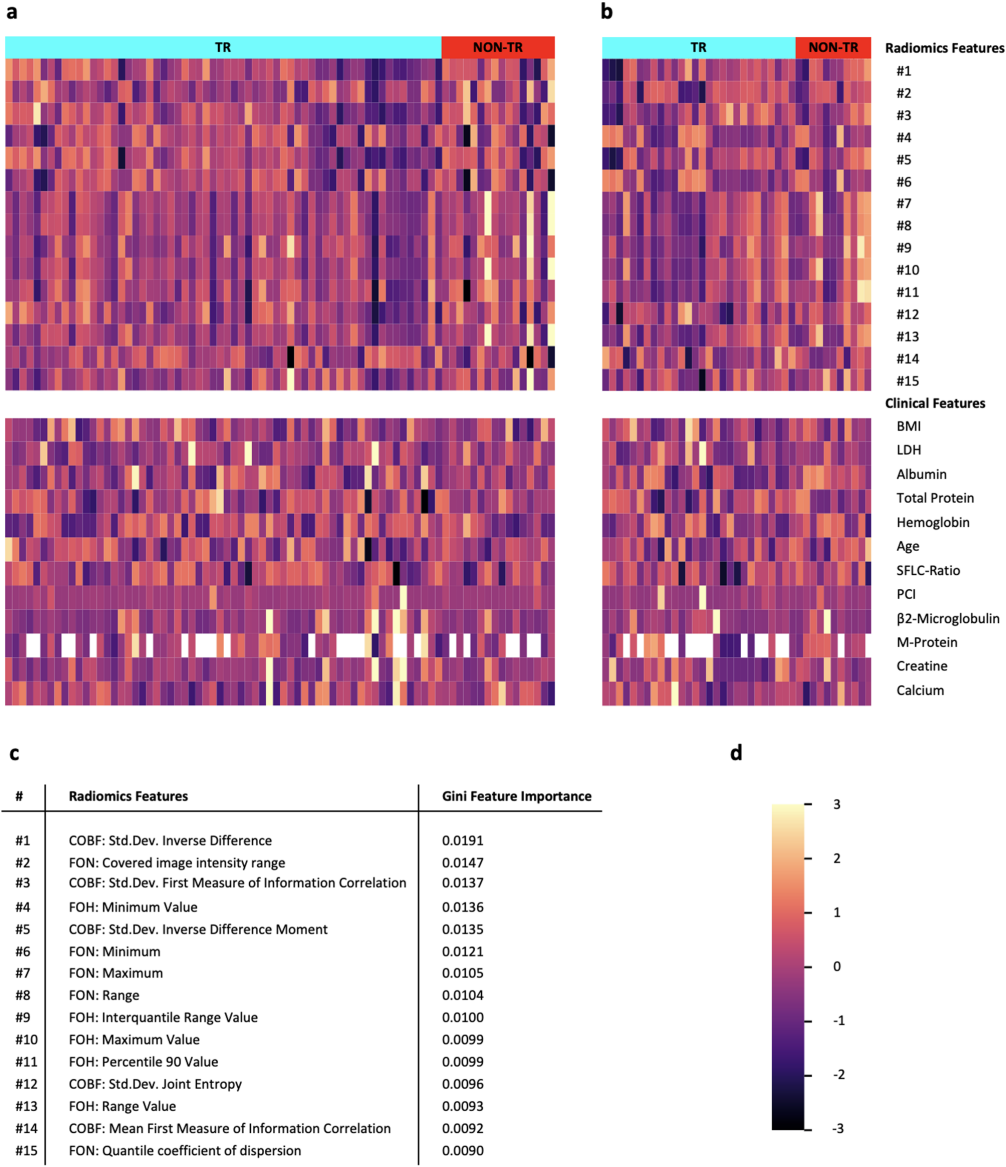
Feature heatmap for the training and test set. Radiomic and clinical signatures of study subjects (given in columns) ordered by TR status in the training set (**a**) and test set (**b**). The 15 most important radiomics features are listed from top to bottom for the radiomics only model (I). The clinical features sex, therapy arm, cytogenetic aberration, and MHC complex are not included due to their categorial configuration. Clinical features with no information have been encoded white. (**c**) The 15 most important individual radiomics features are reported for the radiomics only model (I) by Gini feature importance calculations. (**d**) The color-coded z-score normalization of the clinical and radiomics features are given with standard deviations between − 3 and + 3.

The heatmap presented in Fig. 4 color-encodes the clinical features and the 15 most important radiomics features of the pelvic BM for the radiomics features only model (I). No striking trend can be observed comparing radiomic signatures based on TR binarily classified. Radiomics features contributing most to the respective RFC for TR prediction included features that encode intensity range, maximum and minimum intensity: “first order numeric: maximum”, “first order numeric: range”, “first order histogram: maximum value” and “first order histogram: range value”.

### Prediction of MRD status

For the prediction task MRD negativity, the performance for all radiomics based models (I–III) ranged from 0.52 to 0.54 for the test set. However, models I to III performed worse in absolute terms than the models to predict TR (Table 2; Fig. 3). The prediction performance of the model using clinical features only (IV) resulted in an AUROC of 0.35.

## Discussion

In this study, we developed multiple machine learning models to predict both MRD and TR status based on radiomics features derived from baseline MRI and baseline clinical features. We subsequently investigated their performance on an external, multicentric data set. The algorithmic architecture leveraged a recently presented, nnU-Net-based automated segmentation tool capable of accurate, fully automated pelvic BM segmentation in T1w MRI^31^ allowing a completely automated workflow for radiomics-based prediction. Our results, based on the data from 10 different imaging centers, highlight the predictive value of comprehensive radiomics-encoded information from baseline MRI on TR in MM. The models showed robust performance on external test data, with AUROC values ranging from 0.69 to 0.70 across three radiomics-based model configurations for TR prediction (I-III). These data suggest that predictive radiomics models on baseline MRI in NDMM may provide additional information for clinical decision-making. However, no relevant predictive power could be shown for any of the machine learning models on MRD status.

In a previous study investigating radiomic models for the prediction of TR from baseline MRI, six different ML models were trained with a reported AUROC between 0.80 and 0.89 on an internal test set^25^. Wu and colleagues reported on a radiomics nomogram incorporating the ISS as an independent predictor for the prediction of TR from baseline MRI in a cohort of 123 MM patients, which resulted in an AUROC value of 0.87 compared to a radiomics-only model with an AUROC of 0.86 in their internal test set^26^. Importantly, these prior models were validated internally only, leaving it unclear whether and how the models would generalize in a multicentric clinical application^24,47^. In contrast, our study evaluated prediction performance on a multivendor, multiscanner test set from eight independent centers, providing a realistic estimate of generalizability for potential large-scale clinical deployment.

Also, all earlier models require time-intensive manual segmentation of the lumbar vertebrae before the radiomics features can be extracted, which further undermines future clinical application of said models. In contrast, our algorithmic concept incorporated a previously established nnU-net with a pelvic BM segmentation accuracy equal to that achieved by radiologists^31^. Hence, the all-automated algorithmic concept would allow to implement the presented prediction models in a clinical workflow and imaging platforms to enable scalable, routine use. In the future, patients predicted to have a poor treatment response may benefit from early, personalized cell therapies tailored to their specific disease profile. Reliable baseline prediction of treatment response may therefore contribute to more individualized therapy planning and ultimately improve patient prognosis.

In contrast to TR, our models failed to demonstrate predictive performance for MRD status. This finding differs from study results by Xiong et al., which included 83 MM patients and reported a strong prediction performance with AUROC values of up to 0.84 for their internal test set^27^. However, as with other previous studies, their model lacked external validation and relied on labor-intensive manual segmentation. Moreover, our multicentric design introduces real-world imaging heterogeneity, possibly contributing to more conservative—but clinically relevant—performance estimates.

The difference in predictive performance between TR and MRD may stem from the distinct clinical definitions of the prediction targets. IMWG standard criteria for TR are primarily based on clinical and serological biomarkers, such as m-protein levels and PCI^4^. Radiomics models have previously demonstrated their ability to capture MRI-derived information predictive for these biomarkers, particularly PCI^31^ which could make TR a more accessible and imaging-responsive prediction target. In contrast, MRD assessment allows to detect residual clonal plasma cells with high sensitivity, often revealing disease persistence in patients classified with CR status^4^. MRD negativity thus might represent deeper biological response that may no longer present with imaging features at baseline that prediction models are able to harness automatically, particularly when localized tumor burden is minimal or falls below the threshold of radiological detection. Our findings may therefore underscore the complexity of achieving MRD negativity and the need to include functional imaging modalities in future studies, such as Positron Emission Tomography or diffusion-weighted imaging.

With a median follow up time of 18 weeks from start to end of induction therapy in the underlying GMMG-HD7 study, our study subsequently focused on the prediction of short-term treatment assessment. Baseline clinical parameters associated with MRD and TR after induction therapy as primary/secondary endpoints are limited and have been reported to be only tumor genetics for MRD within the data of the phase 3 GMMG-HD7 trial used in the presented study^28^ and TR^48^.

Our findings underscore the limited performance of prediction models for TR or MRD after induction, if they solely incorporate clinical features. This finding emphasizes the potential of baseline prediction models that are based on, or are complemented by MRI information: Wu et al. reported a significantly worse predictive ability of a purely clinical model for TR, compared to a model that includes radiomics features^26^. For the MRD prediction task, Xiong et al. also found that only the PCI of the BM was associated with MRD status in a univariate analysis, and that a model combining this clinical feature with radiomics features from baseline MRI would show significantly better AUROC results for the prediction of the MRD status than the PCI alone^27^. Following expert recommendation^39^, we included 3 confounders (II) or 16 clinical features (III) in addition to the radiomics features in the prediction models, which lead to a similar prediction performance like the models that relied on radiomics features only (I). These results align with the presented recent studies, indicating that the inclusion of baseline clinical features into radiomics models did not substantially improve the predictive performance for MRD or TR status^26,27,31^.

Our study had several limitations, including its retrospective design. Even though the multicentric data were collected within a randomized controlled phase 3 trial, only a part of the participating patients had received a baseline MRI, resulting in a limited sample size. Radiomics features were extracted from T1w images only, and including information of additional MRI sequences may improve future prediction models. Another limitation is that the volume of interest was limited to the pelvic BM, which would not allow capturing the heterogeneity of MM tumor load distributed over the complete BM and as well as lesions. Recent efforts have explored automated segmentation of the whole-body skeleton^29,49,50^ or diffusion-weighted imaging^30^ which may provide additional information for prediction models through radiomics analysis.

In conclusion, our study showed that automated machine learning models based on radiomics features from baseline MRI have relevant predictive value regarding TR in MM. An independent, multicentric test set comprising data from 8 different centers was used to assess prediction performance. However, the performance of models predicting MRD status was negligible. These results suggest that non-invasive prediction of TR before therapy start could be implementable in the clinical workflow and might improve clinical decision-making. Further studies are warranted to prospectively confirm presented results and to explore whether prediction models can guide therapeutic decisions.

## Supplementary Information

Below is the link to the electronic supplementary material.

Supplementary Material 1

## Data Availability

The data supporting this study’s findings are derived from the ongoing GMMG-HD7 phase-3 trial containing protected patient health information and is subject to EU data protection regulations. Therefore, they are not publicly available. However, data from published portions of the trial may be available upon reasonable request through the corresponding author (FB; fabian.bauer@dkfz-heidelberg.de) to the principal investigator and the board of directors of the GMMG study group, subject to an approved data-sharing agreement and compliance with ethical guidelines.
