# Supplementary material for: Automated radiomics model for prediction of therapy response and minimal residual disease from baseline MRI in multiple myeloma
Source: Sci Rep. 2025 Oct 10;15:35400. doi: 10.1038/s41598-025-13165-2 (PMC12514200; doi:10.1038/s41598-025-13165-2)
Supplement: Supplementary file 1 — Supplementary Material 1 [file 41598_2025_13165_MOESM1_ESM.docx]

**Automated Radiomics Model for Prediction of Therapy Response and Minimal Residual Disease from Baseline MRI in Multiple Myeloma**

**Supplementary Material**

***Authors***

Fabian Bauer^1,2,3+^*, Marina Hajiyianni^4^**^+^**, Niels Weinhold^4^, Martin Grözinger^1^, Jessica Kächele^5,6^, Ekaterina Menis^4^, Marc-Steffen Raab^4^, Sandra Sauer^4^, Anna Jauch^7^, Tim F. Weber^8^, Manuel Debic^8^, Britta Besemer^9^, Marius Horger^10^, Saif Afat^10^, Martin Hoffmann^11^, Johannes Hoffend^12^, Doris Kraemer^13^, Ullrich Graeven^14^, Adrian Ringelstein^15^, Jan Dürig^16^, Lale Umutlu^17^, Heinz-Peter Schlemmer^1^, Hartmut Goldschmidt^4,18^, Klaus Maier-Hein^5,6,18,19^, Stefan Delorme^1^, Elias K. Mai^4^, Markus Wennmann^1,8^**^+^**, Peter Neher^5,6,18,19^**^+^**

**^+^** Contributed equally

* Corresponding author

***Affiliations***

^1^ ﻿Division of Radiology, German Cancer Research Center (DKFZ), 69120 Heidelberg, Germany

^2^ Division of Musculoskeletal Imaging and Intervention, Department of Radiology, Massachusetts General Hospital and Harvard Medical School, Boston, MA 02114, USA

^3^ Institute for Diagnostic and Interventional Radiology, Faculty of Medicine and University Hospital Cologne, University of Cologne, 50937 Cologne, Germany.

^4^ Internal Medicine V, Hematology, Oncology and Rheumatology, Heidelberg University Hospital, 69120 Heidelberg, Germany

^5^ ﻿Division of Medical Image Computing, German Cancer Research Center (DKFZ), 69120 Heidelberg, Germany

^6^ ﻿German Cancer Consortium (DKTK), Partner Site Heidelberg, 69120 Heidelberg, Germany

^7^ Institute of Human Genetics, University of Heidelberg, 69120 Heidelberg, Germany

^8^ Diagnostic and Interventional Radiology, University Hospital Heidelberg, 69120 Heidelberg, Germany

^9^ Department of Hematology, Oncology, and Immunology, University Hospital Tuebingen, 72016 Tübingen, Germany

^10^ Department of Diagnostic and Interventional Radiology, University Hospital Tuebingen, 72016 Tübingen, Germany

^11^ Medical Clinic A, Ludwigshafen Clinical Center, 67063 Ludwigshafen, Germany

^12^ Department of Radiology, Ludwigshafen Clinical Center, 67063 Ludwigshafen, Germany

^13^ Department of Hematology, Oncology and Palliative Care, St. Josefs Hospital Hagen, 58097 Hagen, Germany

^14^ Department of Hematology, Oncology, and Gastroenterology, Kliniken Maria Hilf GmbH, Mönchengladbach, Germany

^15^ Department of Radiology and Neuroradiology, Kliniken Maria Hilf GmbH, 41063 Mönchengladbach, Germany

^16^ MVZ Essen-Nord, St. Augustinus MVZ GmbH, Gelsenkirchen, Germany

^17^ Department of Diagnostic and Interventional Radiology and Neuroradiology, University Hospital Essen, 45141 Essen, Germany

^18^ National Center for Tumor Diseases (NCT), University Hospital Heidelberg, 69120 Heidelberg, Germany

^19^ Pattern Analysis and Learning Group, Department of Radiation Oncology, Heidelberg University Hospital, 69120 Heidelberg, Germany

**Table of Contents**

[**S1. MRI Sequence Parameters** 4](#_Toc193640433)

[**S2. METhodological RadiomICs Score (METRICS)** 5](#_Toc193640434)

[**S3. CheckList for EvaluAtion of Radiomics research (CLEAR)** 7](#_Toc193640435)

[**S4. Calculated Radiomics Features** 10](#_Toc193640436)

[**References** 17](#_Toc193640437)

# **S1. MRI Sequence Parameters**

| **Center (Data Set)**  **Scanner(s) (Field Strength)** | **Coronal T1-Weighted Turbo Spin Echo** | | |
| --- | --- | --- | --- |
|  | **In-Plane Resolution** | **Slice Thickness, Distance Factor** | **Repetition Time, Echo Time** |
| **Center 1** |  |  |  |
| Siemens Magnetom Aera (1.5 Tesla) | 1.3mm x 1.3mm | 5mm, 10% | 528ms/8.4ms |
| **Center 2** |  |  |  |
| Siemens Magnetom Avanto^fit^ (1.5 Tesla) | 1.3mm x 1.3mm | 5mm, 10% | 607ms/6.9ms |
| Siemens Magnetom Aera (1,5 Tesla) | 1.3mm x 1.3mm | 5mm, 10% | 724ms/8.3ms |
| **Center 3** |  |  |  |
| Philips Ingenia (1.5 Tesla) | 1.0mm x 1.0mm | 6mm, 17% | 412ms/4.0ms |
| **Center 4** |  |  |  |
| Philips Ingenia (1.5 Tesla) | 1.0mm x 1.0mm | 6mm, 10% | 351ms/17.5ms |
| **Center 5** |  |  |  |
| Siemens Magnetom Avanto^fit^ (1.5 Tesla) | 1.3mm x 1.3mm | 6mm, 0% | 439ms/11ms |
| Siemens Prisma^fit^ (3.0 Tesla) | 1.3mm x 1.3mm | 6mm, 0% | 439ms/11ms |
| Siemens Magnetom Vida (3.0 Tesla) | 1.3mm x 1.3mm | 5mm, 20% | 400ms/8.4ms |
| **Center 6** |  |  |  |
| Siemens Magnetom Avanto (1,5 Tesla) | 1.3mm x 1.3mm | 5mm, 20% | 627ms/11ms |
| **Center 7** |  |  |  |
| Siemens Magentom Area (1,5 Tesla) | 1.2mm x 1.2mm | 5mm, 10% | 720ms/8.2ms |
| **Center 8** |  |  |  |
| GE Optima MR450w (1.5 Tesla) | 0.9mm x 0.9mm | 8mm, 0% | 429ms/9.0ms |
| **Center 9** |  |  |  |
| Siemens Magnetom Aera (1,5 Tesla) | 0.9mm x 0.9mm | 5mm, 25% | 650ms/8.7ms |
| **Center 10** |  |  |  |
| Philips Intera (1,5 Tesla) | 1.1mm x 1.1mm | 6mm, 0% | 539ms/17.5ms |

**Supplementary Table S1.** List of scanners with respective MRI sequence parameters used for the data sets.

# **S2. METhodological RadiomICs Score (METRICS)**

| **Items/Conditions** | **Definitions** | **Weights** |  |
| --- | --- | --- | --- |
| **Study Design** | | | |
| Item#1 | ? Adherence to radiomics and/or machine learning-specific checklists or guidelines | 0.0368 | yes |
| Item#2 | ? Eligibility criteria that describe a representative study population | 0.0735 | yes |
| Item#3 | ? High-quality reference standard with a clear definition | 0.0919 | yes |
| **Imaging Data** | | | |
| Item#4 | ? Multi-center | 0.0438 | yes |
| Item#5 | ? Clinical translatability of the imaging data source for radiomics analysis | 0.0292 | yes |
| Item#6 | ? Imaging protocol with acquisition parameters | 0.0438 | yes |
| Item#7 | ? The interval between imaging used and reference standard | 0.0292 | yes |
| **Segmentation** | | | |
| Condition#1 | ? Does the study include segmentation? |  | yes |
| Condition#2 | ? Does the study include fully automated segmentation? |  | yes |
| Item#8 | ? Transparent description of segmentation methodology | 0.0337 | yes |
| Item#9 | ? Formal evaluation of fully automated segmentationC | 0.0225 | no |
| Item#10 | ? Test set segmentation masks produced by a single reader or automated tool | 0.0112 | yes |
| **Image Processing and Feature Extraction** | | | |
| Condition#3 | ? Does the study include hand-crafted feature extraction? |  | yes |
| Item#11 | ? Appropriate use of image preprocessing techniques with transparent description | 0.0622 | no |
| Item#12 | ? Use of standardized feature extraction softwareC | 0.0311 | yes |
| Item#13 | ? Transparent reporting of feature extraction parameters, otherwise providing a default configuration statement | 0.0415 | yes |
| **Feature Processing** | | | |
| Condition#4 | ? Does the study include tabular data? |  | yes |
| Condition#5 | ? Does the study include end-to-end deep learning? |  | no |
| Item#14 | ? Removal of non-robust featuresC | 0.0200 | no |
| Item#15 | ? Removal of redundant featuresC | 0.0200 | yes |
| Item#16 | ? Appropriateness of dimensionality compared to data sizeC | 0.0300 | yes |
| Item#17 | ? Robustness assessment of end-to-end deep learning pipelinesC | 0.0200 | n/a |
| Preparation for Modeling |  |  |  |
| Item#18 | ? Proper data partitioning process | 0.0599 | yes |
| Item#19 | ? Handling of confounding factors | 0.0300 | yes |
| **Metrics and Comparison** | | | |
| Item#20 | ? Use of appropriate performance evaluation metrics for task | 0.0352 | yes |
| Item#21 | ? Consideration of uncertainty | 0.0234 | yes |
| Item#22 | ? Calibration assessment | 0.0176 | no |
| Item#23 | ? Use of uni-parametric imaging or proof of its inferiority | 0.0117 | yes |
| Item#24 | ? Comparison with a non-radiomic approach or proof of added clinical value | 0.0293 | no |
| Item#25 | ? Comparison with simple or classical statistical models | 0.0176 | no |
| **Testing** | | | |
| Item#26 | ? Internal testing | 0.0375 | no |
| Item#27 | ? External testing | 0.0749 | yes |
| **Open Science** | | | |
| Item#28 | ? Data availability | 0.0075 | no |
| Item#29 | ? Code availability | 0.0075 | no |
| Item#30 | ? Model availability | 0.0075 | no |
| **Total METRICS score: 76.6%** | | | |
| **Quality category: Good** | | | |

**Supplementary Table S2.** METhodological RadiomICs Score (METRICS) results [1].

# **S3. CheckList for EvaluAtion of Radiomics research (CLEAR)**

| **Section** | **No.** | **Item** | **Yes** | **No** | **n/a** | **Page** |
| --- | --- | --- | --- | --- | --- | --- |
| **Title** | | | | | | |
|  | 1 | ? Relevant title, specifying the radiomic methodology | x |  |  |  |
| **Abstract** | | | | | | |
|  | 2 | ? Structured summary with relevant information | x |  |  |  |
| **Keywords** | | | | | | |
|  | 3 | ? Relevant keywords for radiomics | x |  |  |  |
| **Introduction** | | | | | | |
|  | 4 | ? Scientific or clinical background | x |  |  |  |
|  | 5 | ? Rationale for using a radiomic approach | x |  |  |  |
|  | 6 | ? Study objective(s) | x |  |  |  |
| **Method** | | | | | | |
| *Study design* | 7 | ? Adherence to guidelines or checklists (e.g., CLEAR checklist) | x |  |  |  |
|  | 8 | ? Ethical details (e.g., approval, consent, data protection) | x |  |  |  |
|  | 9 | ? Sample size calculation |  |  | x |  |
|  | 10 | ? Study nature (e.g., retrospective, prospective) | x |  |  |  |
|  | 11 | ? Eligibility criteria | x |  |  |  |
|  | 12 | ? Flowchart for technical pipeline | x |  |  |  |
| *Data* | 13 | ? Data source (e.g., private, public) | x |  |  |  |
|  | 14 | ? Data overlap | x |  |  |  |
|  | 15 | ? Data split methodology | x |  |  |  |
|  | 16 | ? Imaging protocol (i.e., image acquisition and processing) | x |  |  |  |
|  | 17 | ? Definition of non-radiomic predictor variables | x |  |  |  |
|  | 18 | ? Definition of the reference standard (i.e., outcome variable) | x |  |  |  |
| *Segmentation* | 19 | ? Segmentation strategy | x |  |  |  |
|  | 20 | ? Details of operators performing segmentation | x |  |  |  |
| *Pre-processing* | 21 | ? Image pre-processing details |  | x |  |  |
|  | 22 | ? Resampling method and its parameters | x |  |  |  |
|  | 23 | ? Discretization method and its parameters |  | x |  |  |
|  | 24 | ? Image types (e.g., original, filtered, transformed) |  | x |  |  |
| *Feature extraction* | 25 | ? Feature extraction method | x |  |  |  |
|  | 26 | ? Feature classes | x |  |  |  |
|  | 27 | ? Number of features | x |  |  |  |
|  | 28 | ? Default configuration statement for remaining parameters |  | x |  |  |
| *Data preparation* | 29 | ? Handling of missing data | x |  |  |  |
|  | 30 | ? Details of class imbalance | x |  |  |  |
|  | 31 | ? Details of segmentation reliability analysis |  |  | x |  |
|  | 32 | ? Feature scaling details (e.g., normalization, standardization) |  |  | x |  |
|  | 33 | ? Dimension reduction details | x |  |  |  |
| *Modeling* | 34 | ? Algorithm details | x |  |  |  |
|  | 35 | ? Training and tuning details | x |  |  |  |
|  | 36 | ? Handling of confounders | x |  |  |  |
|  | 37 | ? Model selection strategy |  |  | x |  |
| *Evaluation* | 38 | ? Testing technique (e.g., internal, external) | x |  |  |  |
|  | 39 | ? Performance metrics and rationale for choosing | x |  |  |  |
|  | 40 | ? Uncertainty evaluation and measures (e.g., confidence intervals) | x |  |  |  |
|  | 41 | ? Statistical performance comparison (e.g., DeLong's test) | x |  |  |  |
|  | 42 | ? Comparison with non-radiomic and combined methods | x |  |  |  |
|  | 43 | ? Interpretability and explainability methods | x |  |  |  |
| **Results** | | | | | | |
|  | 44 | ? Baseline demographic and clinical characteristics | x |  |  |  |
|  | 45 | ? Flowchart for eligibility criteria | x |  |  |  |
|  | 46 | ? Feature statistics (e.g., reproducibility, feature selection) | x |  |  |  |
|  | 47 | ? Model performance evaluation | x |  |  |  |
|  | 48 | ? Comparison with non-radiomic and combined approaches | x |  |  |  |
| **Discussion** | | | | | | |
|  | 49 | ? Overview of important findings | x |  |  |  |
|  | 50 | ? Previous works with differences from the current study | x |  |  |  |
|  | 51 | ? Practical implications | x |  |  |  |
|  | 52 | ? Strengths and limitations (e.g., bias and generalizability issues) | x |  |  |  |
| **Open Science** | | | | | | |
| *Data availability* | 53 | ? Sharing images along with segmentation data [n/e] |  | x |  |  |
|  | 54 | ? Sharing radiomic feature data |  | x |  |  |
| *Code availability* | 55 | ? Sharing pre-processing scripts or settings |  | x |  |  |
|  | 56 | ? Sharing source code for modeling |  | x |  |  |
| *Model availability* | 57 | ? Sharing final model files |  | x |  |  |
|  | 58 | ? Sharing a ready-to-use system [n/e] |  | x |  |  |

**Table 3**. CheckList for EvaluAtion of Radiomics research (CLEAR) [2].

# **S4. Calculated Radiomics Features**

| **Radiomics Feature** |
| --- |
| Co-occurenced Based Features::Std.Dev. Inverse Difference |
| First Order Numeric::Covered image intensity range |
| Co-occurenced Based Features::Std.Dev. First Measure of Information Correlation |
| First Order Histogram::Minimum Value |
| Co-occurenced Based Features::Std.Dev. Inverse Difference Moment |
| First Order Numeric::Minimum |
| First Order Histogram::Percentile 90 Value |
| First Order Numeric::Maximum |
| First Order Histogram::Interquantile Range Value |
| First Order Histogram::Range Value |
| Co-occurenced Based Features::Mean First Measure of Information Correlation |
| First Order Histogram::Maximum Value |
| First Order Numeric::Range |
| Co-occurenced Based Features::Std.Dev. Joint Entropy |
| First Order Numeric::Quantile coefficient of dispersion |
| Intensity Volume Histogram::Intensity at 0.10 volume |
| Run Length::Run length nonuniformity normalized Std. |
| Run Length::Run percentage Std. |
| Co-occurenced Based Features::Std.Dev. Autocorrelation |
| Run Length::Run length variance Std. |
| Run Length::Run length variance Means |
| Run Length::High grey level run emphasis Std. |
| Co-occurenced Based Features::Overall First Measure of Information Correlation |
| Run Length::Long run emphasis Std. |
| Run Length::Number of runs Std. |
| Co-occurenced Based Features::Std.Dev. Sum Entropy |
| Intensity Volume Histogram::Intensity at 0.90 volume |
| Run Length::Run length variance Comb. |
| First Order Histogram::Robust Mean Absolute Deviation Value |
| Run Length::Short run emphasis Std. |
| First Order Histogram::Quantile coefficient of Dispersion Index |
| First Order Numeric::85th Percentile |
| Co-occurenced Based Features::Std.Dev. Row Average |
| First Order Histogram::Median Absolute Deviation Value |
| First Order Histogram::Variance Value |
| First Order Numeric::Median absolute deviation |
| First Order Numeric::30th Percentile |
| First Order Numeric::95th Percentile |
| First Order Numeric::Coefficient of variation |
| Run Length::Long run emphasis Comb. |
| Run Length::Grey level variance Std. |
| First Order Histogram::Coefficient of Variation Value |
| Co-occurenced Based Features::Std.Dev. Row Entropy |
| Co-occurenced Based Features::Std.Dev. Joint Average |
| First Order Numeric::35th Percentile |
| First Order Numeric::90th Percentile |
| First Order Numeric::Robust mean absolute deviation |
| First Order Numeric::Standard Deviation |
| First Order Histogram::Coefficient of Variation Index |
| First Order Histogram::Quantile coefficient of Dispersion Value |
| Grey Level Size Zone::Zone Size Variance |
| First Order Numeric::80th Percentile |
| Run Length::Long run emphasis Means |
| First Order Numeric::Variance |
| Co-occurenced Based Features::Std.Dev. Sum Average |
| Co-occurenced Based Features::Std.Dev. First Row-Column Entropy |
| Co-occurenced Based Features::Std.Dev. Difference Entropy |
| First Order Numeric::Energy |
| Co-occurenced Based Features::Std.Dev. Second Row-Column Entropy |
| First Order Numeric::45th Percentile |
| First Order Numeric::Mean absolute deviation |
| Grey Level Size Zone::Large Zone Emphasis |
| First Order Numeric::40th Percentile |
| Intensity Volume Histogram::Difference intensity at 0.10 and 0.90 volume |
| First Order Histogram::Minimum Index |
| First Order Histogram::Mean Absolute Deviation Value |
| First Order Histogram::Percentile 10 Value |
| First Order Histogram::Excess Kurtosis Value |
| First Order Numeric::Robust variance |
| Run Length::Run length entropy Std. |
| First Order Numeric::Mode value |
| First Order Histogram::Mode Value |
| First Order Numeric::Kurtosis |
| Grey Level Size Zone::Grey Level Non-Uniformity |
| Grey Level Size Zone::Large Zone Low Grey Level Emphasis |
| First Order Numeric::50th Percentile |
| Co-occurenced Based Features::Overall Correlation |
| First Order Numeric::Interquantile range |
| First Order Numeric::10th Percentile |
| Co-occurenced Based Features::Mean Correlation |
| Co-occurenced Based Features::Overall Joint Maximum |
| First Order Numeric::Excess kurtosis |
| First Order Numeric::05th Percentile |
| Co-occurenced Based Features::Mean Joint Maximum |
| Co-occurenced Based Features::Std.Dev. Inverse Variance |
| First Order Numeric::20th Percentile |
| First Order Numeric::Mean |
| First Order Numeric::70th Percentile |
| First Order Numeric::Median |
| Run Length::Run percentage Means |
| First Order Histogram::Maximum Gradient |
| First Order Numeric::65th Percentile |
| First Order Numeric::Root mean square |
| Grey Level Size Zone::Large Zone High Grey Level Emphasis |
| First Order Histogram::Median Value |
| First Order Histogram::Robust Mean Value |
| First Order Histogram::Excess Kurtosis Index |
| First Order Histogram::Range Index |
| First Order Numeric::Robust mean |
| First Order Histogram::Mean Value |
| Run Length::Run percentage Comb. |
| First Order Numeric::25th Percentile |
| Run Length::Short run emphasis Means |
| Co-occurenced Based Features::Overall Second Measure of Information Correlation |
| First Order Numeric::Skewness |
| Run Length::Long run high grey level emphasis Std. |
| Run Length::Number of runs Means |
| First Order Histogram::Skewness Value |
| Run Length::Number of runs Comb. |
| Run Length::Short run high grey level emphasis Std. |
| Run Length::Run length nonuniformity Comb. |
| First Order Numeric::15th Percentile |
| First Order Histogram::Skewness Index |
| Grey Level Size Zone::Grey Level Non-Uniformity Normalized |
| Run Length::Long run low grey level emphasis Std. |
| First Order Numeric::75th Percentile |
| First Order Histogram::Maximum Index |
| Run Length::Run length nonuniformity normalized Comb. |
| Run Length::Run length nonuniformity Means |
| Co-occurenced Based Features::Std.Dev. Joint Variance |
| Run Length::Run length nonuniformity Std. |
| First Order Numeric::55th Percentile |
| Run Length::Run length nonuniformity normalized Means |
| Co-occurenced Based Features::Overall Inverse Difference Moment |
| Co-occurenced Based Features::Mean Second Measure of Information Correlation |
| Co-occurenced Based Features::Mean Cluster Shade |
| Run Length::Long run low grey level emphasis Means |
| Run Length::Long run low grey level emphasis Comb. |
| Co-occurenced Based Features::Std.Dev. Row Variance |
| Run Length::Low grey level run emphasis Means |
| Co-occurenced Based Features::Std.Dev. Joint Maximum |
| Co-occurenced Based Features::Std.Dev. Correlation |
| Co-occurenced Based Features::Overall Cluster Shade |
| Run Length::Short run emphasis Comb. |
| Run Length::Low grey level run emphasis Comb. |
| Grey Level Size Zone::Zone Size Entropy |
| Co-occurenced Based Features::Overall Inverse Variance |
| Co-occurenced Based Features::Std.Dev. Cluster Shade |
| First Order Numeric::60th Percentile |
| First Order Histogram::Minimum Gradient Index |
| Co-occurenced Based Features::Mean Inverse Difference Moment |
| Run Length::Grey level nonuniformity Std. |
| Grey Level Size Zone::Zone Percentage |
| Co-occurenced Based Features::Std.Dev. Cluster Prominence |
| Co-occurenced Based Features::Mean Difference Variance |
| Co-occurenced Based Features::Std.Dev. Second Measure of Information Correlation |
| First Order Histogram::Mode Index |
| First Order Histogram::Robust Mean Index |
| Co-occurenced Based Features::Mean Inverse Difference |
| First Order Numeric::Mode probability |
| Run Length::Long run high grey level emphasis Means |
| Grey Level Size Zone::Grey Level Variance |
| Co-occurenced Based Features::Mean Inverse Variance |
| First Order Numeric::Mode index |
| Co-occurenced Based Features::Overall Sum Average |
| Co-occurenced Based Features::Overall Joint Average |
| Co-occurenced Based Features::Overall Inverse Difference Normalized |
| Intensity Volume Histogram::Difference volume fraction at 0.10 and 0.90 intensity |
| Co-occurenced Based Features::Mean Joint Average |
| First Order Histogram::Minimum Gradient |
| Co-occurenced Based Features::Mean Difference Average |
| Grey Level Size Zone::Low Grey Level Emphasis |
| Grey Level Size Zone::Zone Size Non-Uniformity Normalized |
| Grey Level Size Zone::Zone Size Mean |
| Run Length::Long run high grey level emphasis Comb. |
| Co-occurenced Based Features::Overall Cluster Prominence |
| Co-occurenced Based Features::Mean Sum Average |
| Co-occurenced Based Features::Overall Contrast |
| Run Length::Grey level nonuniformity Means |
| Run Length::Grey level nonuniformity Comb. |
| Co-occurenced Based Features::Mean Dissimilarity |
| Co-occurenced Based Features::Overall Autocorrelation |
| Co-occurenced Based Features::Overall Difference Variance |
| Co-occurenced Based Features::Overall Joint Entropy |
| Co-occurenced Based Features::Overall Row Average |
| Co-occurenced Based Features::Mean Inverse Difference Normalized |
| Intensity Volume Histogram::Area under IVH curve |
| Co-occurenced Based Features::Mean Row Average |
| Co-occurenced Based Features::Mean Autocorrelation |
| First Order Histogram::Mean Index |
| Co-occurenced Based Features::Std.Dev. Difference Variance |
| Co-occurenced Based Features::Mean Difference Entropy |
| Co-occurenced Based Features::Overall Difference Entropy |
| Grey Level Size Zone::Small Zone Emphasis |
| Co-occurenced Based Features::Std.Dev. Difference Average |
| Co-occurenced Based Features::Overall Row Maximum |
| First Order Histogram::Median Index |
| Intensity Volume Histogram::Volume fraction at 0.10 intensity |
| Co-occurenced Based Features::Mean Cluster Prominence |
| Co-occurenced Based Features::Std.Dev. Sum Variance |
| First Order Histogram::Maximum Gradient Index |
| Co-occurenced Based Features::Std.Dev. Contrast |
| Run Length::High grey level run emphasis Means |
| Co-occurenced Based Features::Mean Joint Entropy |
| Co-occurenced Based Features::Mean Contrast |
| Co-occurenced Based Features::Overall Inverse Difference |
| Co-occurenced Based Features::Overall Difference Average |
| Co-occurenced Based Features::Mean Row Maximum |
| Co-occurenced Based Features::Std.Dev. Dissimilarity |
| First Order Histogram::Percentile 90 Index |
| Co-occurenced Based Features::Overall Dissimilarity |
| Run Length::High grey level run emphasis Comb. |
| Grey Level Size Zone::High Grey Level Emphasis |
| Co-occurenced Based Features::Mean Sum Variance |
| Grey Level Size Zone::Zone Size Non-Uniformity |
| Co-occurenced Based Features::Std.Dev. Cluster Tendency |
| Co-occurenced Based Features::Overall Row Variance |
| Run Length::Short run high grey level emphasis Comb. |
| Co-occurenced Based Features::Overall Cluster Tendency |
| Run Length::Grey level nonuniformity normalized Comb. |
| First Order Histogram::Uniformity Value |
| Co-occurenced Based Features::Mean Cluster Tendency |
| Co-occurenced Based Features::Mean Angular Second Moment |
| Co-occurenced Based Features::Overall Sum Variance |
| First Order Histogram::Percentile 10 Index |
| First Order Histogram::Robust Mean Absolute Deviation Index |
| Co-occurenced Based Features::Mean Joint Variance |
| Co-occurenced Based Features::Mean Row Variance |
| Grey Level Size Zone::Small Zone High Grey Level Emphasis |
| First Order Histogram::Entropy Index |
| Run Length::Grey level nonuniformity normalized Means |
| First Order Numeric::Uniformtiy |
| First Order Histogram::Uniformity Index |
| Run Length::Short run high grey level emphasis Means |
| Co-occurenced Based Features::Overall Angular Second Moment |
| Run Length::Run length entropy Comb. |
| First Order Histogram::Variance Index |
| Co-occurenced Based Features::Mean Sum Entropy |
| Grey Level Size Zone::Grey Level Mean |
| Co-occurenced Based Features::Overall Second Row-Column Entropy |
| Co-occurenced Based Features::Mean Row Entropy |
| Co-occurenced Based Features::Overall Row Entropy |
| Co-occurenced Based Features::Overall Joint Variance |
| Run Length::Grey level variance Comb. |
| Co-occurenced Based Features::Overall Sum Entropy |
| First Order Histogram::Entropy Value |
| Co-occurenced Based Features::Mean Second Row-Column Entropy |
| First Order Numeric::Entropy |
| Co-occurenced Based Features::Overall First Row-Column Entropy |
| First Order Histogram::Mean Absolute Deviation Index |
| Run Length::Grey level variance Means |
| Co-occurenced Based Features::Mean First Row-Column Entropy |
| Run Length::Run length entropy Means |
| First Order Histogram::Interquantile Range Index |
| First Order Histogram::Median Absolute Deviation Index |

**Supplementary Table S2.** List of the calculated radiomics features used as an input for the random forest classifier in the prediction models.

# **References**

1. Kocak B, Akinci D'Antonoli T, Mercaldo N, et al. METhodological RadiomICs Score (METRICS): a quality scoring tool for radiomics research endorsed by EuSoMII. *Insights Imaging* 2024; 15:8

2. Kocak B, Baessler B, Bakas S, et al. CheckList for EvaluAtion of Radiomics research (CLEAR): a step-by-step reporting guideline for authors and reviewers endorsed by ESR and EuSoMII. *Insights Imaging* 2023; 14:75
